# Multistability of the Brain Network for Self-other Processing

**DOI:** 10.1038/srep43313

**Published:** 2017-03-03

**Authors:** Yi-An Chen, Tsung-Ren Huang

**Affiliations:** 1 Department of Psychology, National Taiwan University, 10617 Taipei, Taiwan

## Abstract

Early fMRI studies suggested that brain areas processing self-related and other-related information were highly overlapping. Hypothesising functional localisation of the cortex, researchers have tried to locate “self-specific” and “other-specific” regions within these overlapping areas by subtracting suspected confounding signals in task-based fMRI experiments. Inspired by recent advances in whole-brain dynamic modelling, we instead explored an alternative hypothesis that similar spatial activation patterns could be associated with different processing modes in the form of different synchronisation patterns. Combining an automated synthesis of fMRI data with a presumption-free diffusion spectrum image (DSI) fibre-tracking algorithm, we isolated a network putatively composed of brain areas and white matter tracts involved in self-other processing. We sampled synchronisation patterns from the dynamical systems of this network using various combinations of physiological parameters. Our results showed that the self-other processing network, with simulated gamma-band activity, tended to stabilise at a number of distinct synchronisation patterns. This phenomenon, termed “multistability,” could serve as an alternative model in theorising the mechanism of processing self-other information.

Social neuroscientists have been trying to identify brain areas processing self-related and other-related information. These brain areas might be affected in autism, major depressive disorder and schizophrenia, which were associated with abnormal self-referential processing or mental state inference^1^,^2^,^3^. In many studies of functional magnetic resonance imaging (fMRI), brain areas activated by self-related or other-related tasks were contrasted against each other, or against some factors that were considered confounders of the relationships being studied, such as familiarity or closeness^4^,^5^,^6^. However, accumulating imaging data over the last decade still showed a high degree of overlap between self-related and other-related brain areas^7^. Although the overlapping areas could be interpreted as yet-to-be-subtracted confounding signals according to the functional localisation hypothesis of fMRI, recent advances in neural network dynamics have cast doubt on the essentialness of the localisation hypothesis.

Is it possible that two distinct concepts—self and other, for example—are processed by the same set of brain areas, hence similar activation patterns? Earlier ideas were conceived in studies of “mirror neuron,” which was found to respond similarly to self-initiated and other-initiated movements^8^, yet the interpretation was still debated^9^,^10^. Recent studies have shed light on the idea from a different perspective. Hansen *et al*. conducted human whole-brain computational modelling based on white matter connections derived from DSI and physiologically grounded neural models^11^. Compatible with empirical data, the simulations demonstrated existence of multiple distinct whole-brain synchronisation patterns that were characterised by distinct epochs along the evolution trajectory of the same functional connectivity dynamics. More importantly, their data showed that synchronisation patterns were not bound to spatial activation patterns. In other words, synchronisation patterns could differ even when fMRI spatial patterns appeared similar. Some researchers have proposed to integrate the dynamic synchronisation theory developed by simulation studies with task-induced changes in selective coherence observed in task-based experiments^12^. In summary, loosening the functional localisation constraints and incorporating dynamic properties revealed by simulation studies may help researchers gain more insight into information processing.

This type of structural-connection-based dynamic computational modelling has not yet been applied to localised brain areas recognised by task-based fMRI as pertaining to particular cognitive functions. The reason might be the seeming incompatibility between these two approaches. Dynamic computational modelling, which concerned itself with properties at the network level, generally avoided unwarranted exclusion of any brain area, since it might perturb the network configuration^13^. Therefore, most studies were conducted at the whole-brain level^11^,^14^,^15^. Besides, the nodes—basic computing unit in a model—were usually defined by anatomical landmarks or parcellation schemes that segmented the brain into a few dozens to hundreds of cortical regions^13^,^16^,^17^. The degree of fineness of these parcellation schemes might be appropriate at the whole-brain level, but probably too low to capture the network structure of more confined brain areas. Despite of these conceptual and technical issues, it was generally accepted that brain areas supporting cognitive functions were spatially segregated to some extent, and the balance between integration and segregation has become an intensely investigated topic^18^. If the tendency of shifting between multiple stable synchronisation patterns—referred to as “multistability”—can be verified in a structural brain network whose cortical regions constitute cognitive modules identified by task-based fMRI, the cognitive implication of multistability can draw more support.

In the present study, we modified the dynamic computational modelling method to make it better suited for localised neural networks while complying with the principles of node definition in network topology analysis^19^,^20^. Combining fMRI term-based meta-analysis and DSI fibre tracking, we constructed a brain network by tracing out all fibre connections between brain areas putatively engaged in self-other processing. Next, we sampled synchronisation patterns from structural-connection-based dynamical systems to characterise the dynamic organising properties of this brain network. For methodological comparison, the same simulation procedure was conducted on two separate brain networks: One with an anatomical parcellation scheme, the other with a random parcellation scheme with matched node number. We hypothesised that the self-other processing network in the brain could present multistability, which engendered dynamic shifting between different information-processing modes.

## Results

### Self-other processing areas and their structural connectivity

**Figure 1:**
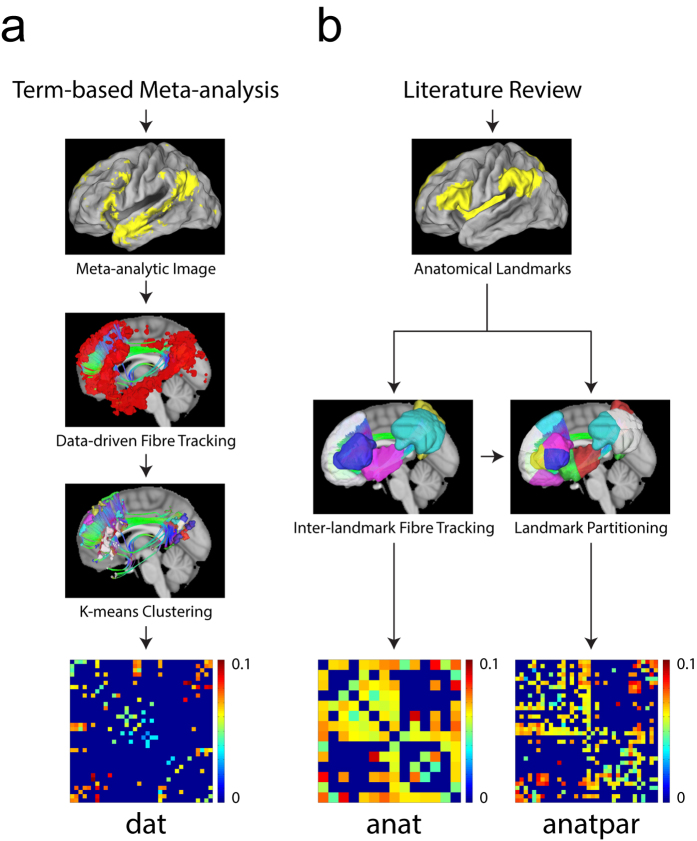
Self-other processing networks constructed by three different methods. (**a**) The image derived from Neurosynth term-based meta-analysis was registered to the NTU-122 DSI template, after which fibre tracking and k-means clustering were used to construct structural connections between any two regions within this image and define network nodes. The structural connectivity matrix of this *dat* network was shown at the bottom. Colour bar unit: Generalised Fractional Anisotropy (GFA). (**b**) 14 anatomical regions relevant to self-other processing were registered to the NTU-122 template. Fibre connections were constructed between all pairs of regions, giving rise to the *anat* network (bottom left). The *anatpar* network had the same white matter structure as *anat* while its nodes were composed of 34 random segments rather than the original 14 anatomical areas (bottom right).

The self-other processing network, which should include brain areas and white matter tracts putatively engaged in self-related and other-related information processing tasks, was constructed with a data-driven approach. The process was depicted in the flow chart in Fig. 1a. First, we conducted a term-based meta-analysis by the Neurosynth tools and database to identify brain areas relevant to self-other processing^21^. Spatial coordinates reported in fMRI studies tagged by the key terms “self referential,” “mind tom,” “theory mind” and “mentalizing” were extracted from a database containing more than 10,900 fMRI studies, and a chi-square test was conducted to identify coordinates whose occurrence frequencies in the tagged studies were significantly higher than untagged studies. The meta-analytic images for “self” and “other” were shown in Supplementary Fig. S1. The result of a two-way chi-square analysis of these two groups of studies conducted by Neurosynth tools revealed no statistically significant difference between “self” and “other” coordinate distributions. These two images were merged into a complete “self-other processing” image for subsequent DSI fibre tracking. It should be mentioned that meta-analytic results produced by Neurosynth might not be as specific as traditional meta-analytic methods. However, we actually preferred a meta-analytic image that was more encompassing rather than narrow and specific, because social neuroscientists had not yet reached consensus on the operational definition of “self-processing” and “other-processing”^22^,^23^. Therefore, setting strict criteria on fMRI task types might bias the meta-analytic image toward certain types of tasks, hence biasing the definition of self-related and other-related information processing.

Next, the meta-analytic image was registered to a normalised DSI template to explore the fibre connections between self-other processing brain areas. As stated in the Introduction, task-related brain areas usually account for only a small part of the cerebral cortex, making their network configurations particularly prone to variations caused by differences in cortex parcellation schemes and the selection of regions of interest (ROI). Therefore, we tried to minimise *a priori* anatomical constraints imposed on fibre tracking algorithm by employing a data-driven approach. Instead of establishing major white matter tracts between pairs of pre-defined cortical segments^24^,^25^,^26^, the fibre-tracking algorithm was adjusted for a free exploration of all fibre connections between any two voxels located within the meta-analytic image. After tracing out all connections between any two regions within the meta-analytic image, we assigned nodes to the network by a k-means clustering analysis of the fibre end points. Voxels containing spatially close end points of fibres with similar orientations were spontaneously grouped into one node, which satisfied the node definition of spatial proximity and connectional homogeneity in network topology studies^19^,^20^. The resulting network, termed ‘*dat*’, and its connectivity matrix, was shown in the bottom of Fig. 1a.

As mentioned in the Introduction, previous studies of network analysis usually involved *a priori* definition of nodes by various parcellation schemes. Although we did not adopt this approach to establish *dat* network, a methodological comparison was necessary. Therefore, we constructed a separate network by establishing white matter tracts between pre-defined anatomical areas that were considered relevant to self-other processing in the literature. After a thorough literature review, the following anatomical landmarks were included: Anterior cingulate cortex, posterior cingulate cortex, precuneus, medial prefrontal cortex, pars triangularis and pars opercularis of inferior frontal gyrus, temporo-parietal junction, and insula^5^,^7^,^27^,^28^,^29^. These landmarks were manually traced out and registered to the DSI template, after which fibre tracts confined to these anatomical landmarks were constructed. This *anat* network and its connectivity matrix, with anatomical landmarks serving as the nodes, were shown at the bottom left of Fig. 1b.

The *anat* network had 14 nodes, while the *dat* network had 34. This raised the concern that network resolution or node number might confound the comparison. Therefore, we constructed the third network, *anatpar*, by randomly partitioning the *anat* nodes into 34 smaller segments that were comparable in size. Essentially, *anatpar* retained the white matter structure of *anat* while the nodes were replaced by 34 smaller random cortical segments. This *anatpar* network was shown at the bottom right of Fig. 1b.

### Neural simulation and synchronisation analysis

We then conducted neural model simulations on the networks, creating oscillatory activities that mimicked gamma oscillations in the brain recorded in experimental settings^12^,^30^. There were many types of neural mass models that aimed at capturing different aspects of neural activities, such as mean field model^31^, linear stochastic model^32^, and Kuramoto model^14^. We chose Kuramoto model because it was physiologically inspired, computationally efficient, and focused on the oscillatory activities. The mathematical derivation and physiological interpretation of Kuramoto model was detailed in earlier literatures^33^,^34^; briefly, this model treated brain areas as weakly coupled oscillators that influenced each other through fibre connections of different lengths and strengths. In the present study, we randomly sampled 200 different combinations of *N* intrinsic frequencies from the gamma range (25 Hz to 75 Hz) for each *N*-node network, making the exploration of physiological parameters as exhaustive as possible. One combination of intrinsic frequencies constituted one unique dynamical system, whose multistability should be assessed independently of other dynamical systems. Thus the 200 dynamical systems derived from the same neural network were assessed one by one, and the overall tendency of multistability constituted the multistable potential of the given brain network.

It should be mentioned that we did not include the noise term in the original equation of Kuramoto model. Noise was a freely adjustable parameter in the model and might play a role in driving the neural network into different synchronisation states. However, the current study aimed to explore the multistable potential granted by certain network structural configurations rather than characterising the detailed evolution along the temporal dimension. Besides, the exploration method we chose depended on the stabilisation of dynamical systems at particular synchronisation states. Therefore, we did not add noise to the Kuramoto model.

**Figure 2:**
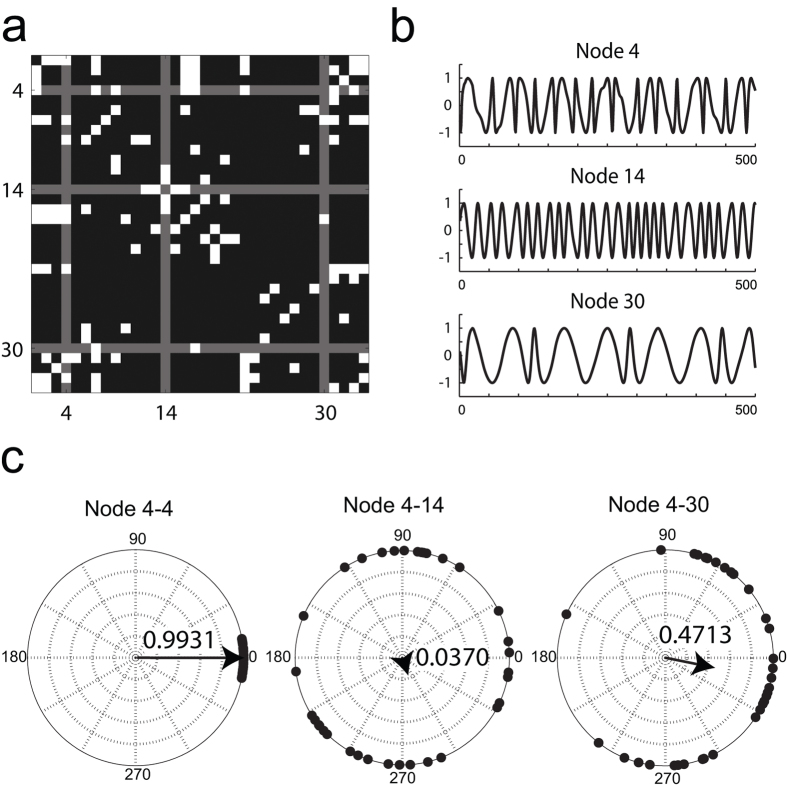
Neural mass model, oscillatory trace simulation and the synchronisation levels. (**a**) Three nodes in the *dat* network were chosen for illustration: Node 4, node 14, and node 30. Columns and rows representing these nodes were highlighted with gray in the connectivity matrix; the presence of fibre connections between any two nodes was indicated by white at their intersection entries. As shown in the matrix, the three nodes were not inter-connected. (**b**) First 500 iterations of Kuramoto-model-simulated oscillatory traces of the three nodes. Horizontal axis: Iteration number. Vertical axis: Amplitude. (**c**) Illustration of synchronisation levels and relative phase delays between pairs of nodes, with node 4 serving as the reference. Dots on the polar coordinates indicated the concurrent phase signals of the non-reference nodes. The left polar graph presented the synchronisation between node 4 and itself; as expected for auto-correlation, the synchronisation level was high (level indicated by vector length and labelled in graph), with nearly no phase delay (phase delay indicated by vector angle). The synchronisation between node 4 and the other two nodes varied in strength and delay (middle and right panels).

All Kuramoto model simulations underwent 2,000 iterations to ensure stabilisation into a particular synchronisation state, and signal correlations between any two nodes at stability were evaluated to obtain the network synchronisation pattern. Three nodes in the *dat* network were highlighted in Fig. 2a for illustration; their simulated Kuramoto signals were presented in Fig. 2b. All Kuramoto signals were mapped to the phase space by Hilbert transform for stroboscopic analysis, which scored the phase correlation between any two nodes with a synchronisation index ranging from 0 to 1. For *dat* network with 34 nodes, each simulation process produced 561 pairwise synchronisation indices, forming the overall synchronisation pattern of a particular simulation at stability. Figure 2c illustrated the concept of stroboscopic analysis: The reference node 4 was fixed at 2π, and the concurrent phases of the other nodes—node 4, 14 and 30—were plotted on their respective polar coordinate systems (Fig. 2c, from left to right). Synchronisation level between any of the plotted nodes with the reference node was reflected by the averaged norm of the sum of unit vectors scattered around its polar coordinate system.

In summary, each of the three networks—*dat, anat, anatpar*—were assigned 200 random combinations of intrinsic frequencies, producing 200 dynamical systems for each. Kuramoto-model simulations and stroboscopic analyses were then conducted on the three groups of dynamical systems to obtain their synchronisation patterns at stable states.

### Multistability analysis

To explore the multistability potential of the three self-other neural networks, their corresponding repertoires of 200 sampled dynamical systems with gamma oscillations were tested individually for multistability, namely the ability to present multiple stable synchronisation patterns. The collective results of the 200 dynamical systems constituted the multistable potential of each corresponding neural network. As explained in the previous section, one simulation yielded one synchronisation pattern. To assess multistability, each dynamical system was simulated for 100 times from randomly selected initial conditions (i.e., oscillation phases), and the 100 synchronisation patterns were sorted by their similarity to show clustering tendency. This treatment was based on the theory that attractor states of a dynamical system could be traced out by tracking the evolution trajectories from different initial conditions. If the evolving trajectories of the dynamical systems converged to certain points within the state space, the points were considered attractors, and the dynamical system was deemed multistable^34^,^35^. On the other hand, if the dynamical system contained only one attractor state or no attractor states at all, the trajectories would either converge to the same point or fail to converge. Both conditions manifested themselves by a lack of clustering tendency of the 100 randomly sampled synchronisation patterns.

**Figure 3:**
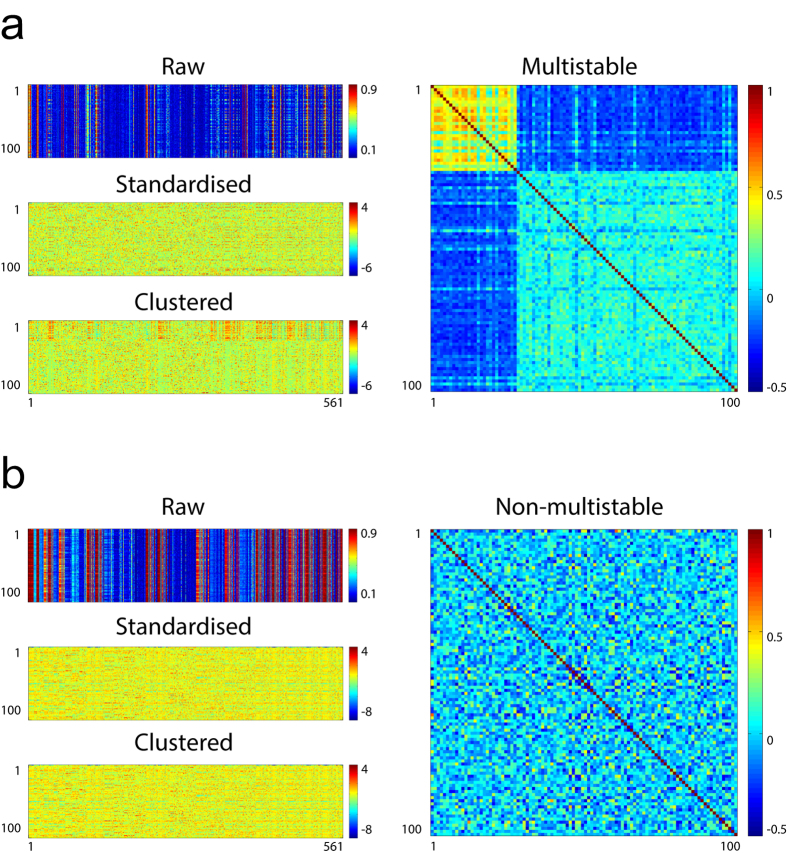
Multistability analysis of individual dynamical systems. (**a**) Analysis of one dynamical system from the *dat* category. *Raw* matrix represented the 100 synchronisation patterns derived from 100 randomly phase-initiated Kuramoto simulations; each row in the matrix represented one synchronisation pattern composed of 561 pairwise correlation indices of the 34 nodes in *dat* network. The matrix was standardised column-wise (*Standardised*) and clustered by the k-means clustering algorithm with the Elbow method (*Clustered*). Note that the 100 synchronisation patterns could be classified into two distinct subtypes in the *Clustered* matrix. The Pearson correlation coefficient matrix (right) of the *Clustered* matrix better visualised the two distinct subtypes as two blocks along the main diagonal. (**b**) Analysis of another dynamical system from the *dat* category. For this case, the k-means clustering algorithm could not identify subtypes within the 100 synchronisation patterns, hence no main-diagonal blocks in the Pearson correlation coefficient matrix.

The above-mentioned analysis was illustrated in Fig. 3, which presented two of the dynamical systems from the *dat* category. The matrix labelled “Raw” in Fig. 3a represented the 100 synchronisation patterns of the particular dynamical system; each row in the matrix—constituted of 561 stroboscopic analysis-derived phase-correlation indices representing the pairwise synchronisation levels of the 34 nodes—stood for one synchronisation pattern. The matrix was then standardised column-wise as a pre-processing step for k-means algorithm^36^, after which k-means clustering with the Elbow method was used to determine whether the 100 synchronisation patterns could be segregated into distinct clusters^37^. For this particular case, the algorithm determined that the synchronisation patterns could be segregated into two dissimilar clusters. For better visualisation, we cross-correlated the “Clustered” matrix with itself to obtain a Pearson correlation coefficient matrix that clearly revealed two distinct modules along the main diagonal. This indicated that the dynamical system was able to stabilise at two distinct types of synchronisation patterns. This particular dynamical system was therefore multistable. On the contrary, the other dynamical system did not show multistability (Fig. 3b).

Basically, any claim of network properties—such as the presence of multistability—should be based on a comparison to null-hypothesis networks, which were constructed by randomising the original networks in a way that preserved the node number, edge number, degree distribution, connectivity strength and length of the original networks^19^. Therefore, we created 15 randomised networks for each of the three networks and repeated the same simulation and analyses on them. The results obtained from randomised networks would be presented along with their corresponding neural networks in the following section.

### Comparison of *dat, anat* and *anatpar* networks

**Figure 4:**
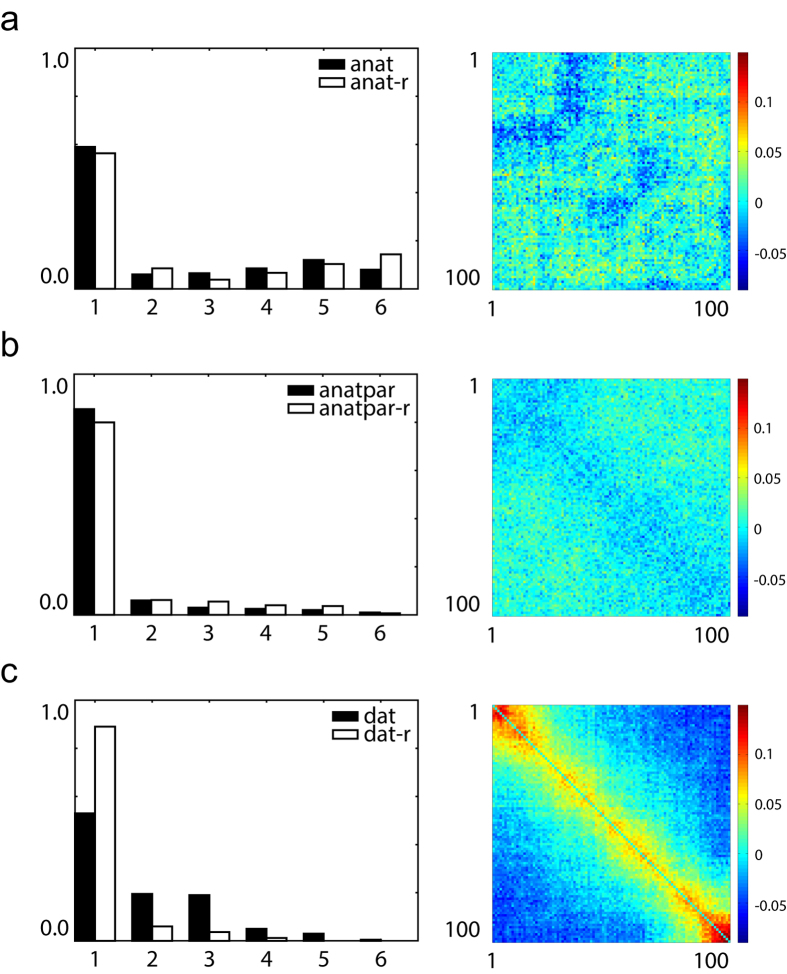
Comparison of multistability potential between three networks. (**a**) Left: Number distributions of stable synchronisation states of *anat* and *anat-r* did not show statistically significant difference (p = 0.5706). Horizontal axis: Number of stable synchronisation states. Vertical axis: Ratio of counts. Right: Difference between average *anat* and *anat-r* pattern-correlation matrices did not reveal aggregation of high-value entries near the main diagonal, indicating lack of multistability. (**b**) Left: Number distributions of stable synchronisation state of *anatpar* and *anatpar-r* did not show statistically significant difference (p = 0.7431). Right: Difference between average *anatpar* and *anatpar-r* correlation matrices did not reveal aggregation of high-value entries near the main diagonal, indicating lack of multistability. (**c**) Left: Number distributions of stable synchronisation states of *dat* and *dat-r* showed statistically significant difference (p = 7.9088 × 10^−18^). Right: Difference between average *dat* and *dat-r* correlation matrices revealed aggregation of high-value entries near the main diagonal, indicating multistability.

After finishing the simulation and analysis described in previous sections, we obtained three groups of multistability assessment results. The results, presented in Fig. 4, were summarised from 200 correlation matrices of gamma-band synchronisation patterns for *dat, anat* and *anatpar* network respectively. The number distributions of stable synchronisation states for each network were presented along with the results obtained from their corresponding random networks (*dat-r, anat-r* and *anatpar-r*). The presented square matrices were created by subtracting the average pattern-correlation matrix of randomised networks from the average pattern-correlation matrix of the three brain networks. These contrast matrices visualised the difference between brain networks and randomised networks in terms of multistability potential; if brain networks tend to be multistable, the matrices should reveal an aggregation of high-value entries near the main diagonal.

Figure 4a presented the results of the *anat* neural network. The number distribution of stable synchronisation states showed that 59% of the 200 dynamical systems did not present multistability; the other 41% near-equally distributed between 2 to 6 stable states. The distribution was not significantly different from its randomised counterparts (two-sided two-sample Kolmogorov-Smirnov test, alpha level = 0.01, p = 0.5706). As a result, the contrast matrix did not show apparent aggregation of high-value entries near the main diagonal, namely multistability.

Figure 4b presented the results of the *anatpar* neural network, a network having the same number of nodes as *dat* network and the same white matter structure as *anat* network. 85.5% of its dynamical systems did not present multistability, and the number distribution of stable synchronisation states was not significantly different from its null-hypothesis networks (two-sided two-sample Kolmogorov-Smirnov test, alpha level = 0.01, p = 0.7431). Consequently, its matrix did not show apparent tendency of multistability.

Figure 4c presented the results of the *dat* neural network. Unlike the other two networks, the distribution of stable synchronisation states from the *dat* network and its corresponding randomised networks were significantly different (two-sided two-sample Kolmogorov-Smirnov test, alpha level = 0.01, p = 7.9088 × 10^−18^); the *dat* distribution was skewed toward the higher stable state numbers compared to the null-hypothesis distribution. Hence, the contrast matrix showed an aggregation of high-value entries near the main diagonal, indicating a tendency of multistability.

We also compared distributions of stable synchronisation states among *dat, anat* and *anatpar*. Two-sided two-sample Kolmogorov-Smirnov test indicated significant differences between *dat* and the other two networks (alpha level = 0.01, p = 5.4331 × 10^−4^ for *anat*; p = 7.6668 × 10^−10^ for *anatpar*).

The comparisons above focused on differences between different network construction methods, and the results suggested that multistability was uniquely associated with the network constructed by data-driven approach. It was therefore important to clarify whether multistability should be attributed to the self-other-related network configuration or the data-driven construction method itself. To answer this question, we independently applied the same network construction method to the “self” and “other” meta-analytic images shown in Supplementary Fig. S1. The *self* network was composed of 17 nodes, and the *other* network was composed of 30 nodes. Kuramoto simulations and multistability analyses were conducted in the same way, and the results were shown in Supplementary Fig. S2. The results suggested that *other* network did not reveal significant multistability. The experiment indicated that the data-driven network construction algorithm did not necessarily lead to the same degree of multistability of any combination of brain areas.

## Discussion

Supported by a wide variety of evidence, functional localisation hypothesis has long been acknowledged in the neuroscience field. It is widely accepted that localised damage at Brodmann area 22 is associated with Wernicke’s aphasia^38^, and removing bilateral hippocampus permanently interrupts episodic memory formation^39^. However, the hypothesis seems to reach its limit when distinct psychological concepts induce similar spatial activation patterns in fMRI experiments. As illustrated in Supplementary Fig. S1, the processing of self-related versus other-related information is one such case, and researchers have tried to resolve this issue by dissembling these psychological processes into more basic components, such as familiarity and mental state attribution^5^,^22^. These components were then manipulated in fMRI experiments in an attempt to isolate brain areas that were “self-specific” or “other-specific.” However, many of these basic components were too abstract for researchers to operationalise, leading to difficulty and inconsistency in the interpretation of fMRI results.

Recent advances in dynamic computational modelling provided a different way to explore this issue. Combining neural simulation and Balloon-Windkessel model, researchers demonstrated the possibility of different synchronising modes exhibiting similar fMRI spatial patterns^11^. Note that such differential couplings among the same set of brain areas through dynamic synchronisation echoed the earlier proposal that brain dynamically engaged different cortical areas through oscillatory coupling to achieve different tasks^30^,^40^. As an example, some researchers applied the dynamic synchronisation theory to interpret discoveries in task-based experiments, such as the selective entrainment of higher visual area V4 by different neuronal groups in lower visual area V1^12^.

Some methodological gaps existed between the simulation studies and the task-based experiments, though. First, while different synchronisation patterns might be associated with similar fMRI spatial patterns, the synchronisation patterns were simulated at the whole-brain level. Therefore, it might be difficult to associate the synchronisation dynamics to regional activities that were implicated in specific cognitive functions. Second, the whole-brain simulation studies were not task-based. In fact, many of these studies compared their results to human resting-state fMRI^11^,^14^,^15^. Third, the brain parcellation schemes used in whole-brain modelling might not be applicable to localised neural networks corresponding to certain cognitive modules, which had smaller spatial scale.

In the present study, we tried to connect dynamic computational modelling to task-based studies by making a few modifications of the original approach. We identified brain areas involved in self-other processing by conducting a term-based meta-analysis using Neurosynth tools and database. Neurosynth is sometimes criticised for its automated text mining and coordinate extraction approach, which raise concerns about its meta-analytic sensitivity and specificity. However, manual meta-analysis may be subject to selection bias, especially when the concept being investigated is abstract. Specifically, traditional meta-analysis of psychological concepts required a clear operational definition, which formed the basis of literature inclusion criteria. However, the operationalisation might involve personal preference on how the abstract concepts—such as “self” and “others”—should be interpreted. In fact, literatures extracted by key words in this study revealed a variety of tasks that researchers claimed to be related to self-processing or other-processing, such as autobiographical memory retrieval, future scene construction, attributing intention, attributing false belief, and gaze processing. There is currently no consensus on whether self-other processing depends on the union or intersection of all these cognitive functions; in this study, we chose union to avoid personal-preference bias. Combining this encompassing meta-analytic image with a DSI fibre-tracking algorithm that minimised anatomical constraints, we supposed that *dat* network included most of the brain areas and white matter tracts implicated in self-other processing, and the definition of nodes in *dat* network complied with the principles of network topology analysis. Simulation results derived from this network suggested that self-other processing network had the potential of presenting multistability.

**Figure 5:**
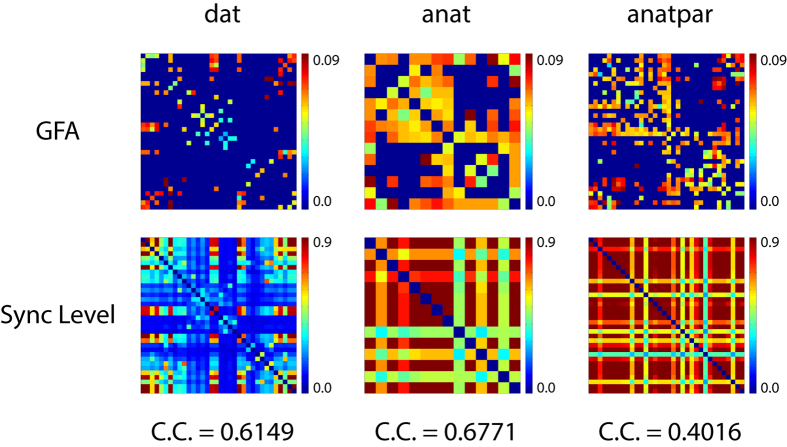
Comparison of GFA patterns and synchronisation patterns of three networks. The top three matrices were the GFA matrices of *dat, anat* and *anatpar* networks. The bottom three matrices were individually created for each network by averaging the 20,000 synchronisation patterns derived from Kuramoto simulation and stroboscopic analysis and mapping these average pair-wise synchronisation values back to a *N* × *N* matrix, with each entry indicating the average pair-wise synchronisation index of a pair of nodes. The Pearson correlation coefficient between the GFA matrix and the synchronisation matrix for each network was listed below each column. C.C.: Correlation coefficient. Sync: Synchronisation pattern.

Earlier studies have suggested a correlation between structural connectivity and static synchronisation pattern^41^. Consistent with those studies, the average synchronisation patterns of all three localised networks in our study showed positive correlation with their structural connectivity patterns (Fig. 5). However, the non-stationarity of functional connectivity demands more sophisticated analysis that considers the dynamic aspects of networks^42^. The functional connectivity dynamics (FCD) analysis proposed by Hansen *et al*. fulfilled such requirement^11^, and the multistability analysis in the present study actually bore some resemblance to the FCD analysis. The main difference was that we did not sample different synchronisation patterns by noise-driven exploration of dynamical system; we applied random sampling of initial conditions on noise-free dynamical systems instead. Unlike FCD, this algorithm was not designed for real fMRI signal analysis, since it assumed the dynamical systems to be noise free. Our multistability analysis could be considered as being built on the foundation laid by FCD study. FCD analysis revealed the dynamic synchronisation phenomenon in empirical brain networks and explained it with the properties of fixed-point attractors in dynamical systems; our multistability analysis identified fixed-point attractors in DSI-based empirical networks with a massive sampling approach, presuming their contribution to multistability. Because multistability analysis concerned itself with the network structural configuration rather than particular parameter settings, the result should be conservatively interpreted as the “potential” of multistability granted by the given network configuration rather than physiologically-verified multistability. Nevertheless, simulation results could be tested in the experimental setting.

With the advance of high-performance computing and rapid accumulation of brain imaging data, the neuroscience field seems to be experiencing a paradigmatic shift from detailed inspection of individual brain areas or tracts to characterisation of emergent features at the system level. Here we tried to find a middle ground between the functional localisation hypothesis and the dynamic synchronisation theory. Illustrating the existence of different synchronisation modes being adopted by the same set of activated brain areas with the controversial case of self-other processing, we explored an information processing mechanism distinct from that illustrated by functional localisation hypothesis. The results and methods proposed in this study might complement other study designs in the exploration of mechanisms underlying the dynamic reconfiguration of brain synchronisation and their cognitive implications.

## Methods

### Structural networks

The dynamical properties of a network depend on both its topological structure and node activities. The topological structure may not be entirely objective, however, because it depends on the construction approach selected. For example, two researchers may construct two versions of attention network that involve the same set of anatomical areas—intra-parietal sulcus, frontal eye field, etc.—yet differ in topological structures, because they use different brain atlases. It was for this reason that we developed a data-driven approach that relied on meta-analyses rather than arbitrary atlases. Nevertheless, it would be better to compare the simulation results with those of networks constructed by conventional approaches, since the properties of this new data-driven approach has not yet been thoroughly investigated. As stated above, there is no “standard” conventional approach, because there is no standard cortex parcellation scheme. In order to make the comparison between data-driven approach and conventional approach more generalisable, two versions of conventional networks—*anat* and *anatpar*—were constructed. These two networks, together with the *dat* network constructed by the data-driven approach, constituted the three structural networks in this study. Their design concepts and detailed construction processes are described below.

The *dat* network—“*dat*” meaning “data-driven”—was the main target of investigation. The goal of constructing *dat* was to investigate the dynamical properties of a self-other processing network constructed by a relatively objective, atlas-independent approach. Three features separated *dat* network from the other two networks. First, seed regions for its fibre-tracking process were derived from meta-analyses rather than arbitrary atlases. Second, we did not segment the seed regions into distinct ROIs and trace fibres between them; instead, we explored all possible connections both “within” and “between” brain regions. Third, network nodes were defined by fibre-endpoint clustering algorithm rather than arbitrary atlases.

The technical details of *dat* construction is described below. It was constructed by tracing out white matter connections between brain areas within a merged meta-analytic image created by the Neurosynth tools and the version 0.5 database. The database contained activation contrasts from more than 10,900 fMRI studies. After a preliminary exploration of a combination of different key words, we chose the key word “self referential” to conduct the term-based meta-analysis for self-related processing, and a combination of “mind tom,” “theory mind,” “mentalizing” minus “self” for other-related processing. The term frequency-inverse document frequency (TF-IDF) was set to 0.05 for both meta-analyses. The false discovery rate (FDR) was set to 0.05; both forward inference and reverse inference images were obtained, and statistical threshold was set to Z = 4.5. The automated literature extraction returned 116 studies for self-related processing and 168 studies for other-related processing. We performed a meta-analytic comparison between the two groups of studies; spatial activation patterns of the two were not significantly different. The union of these meta-analytic images, with subcortical regions removed, was used in DSI fibre tracking. The “self” and “other” meta-analytic images were independently shown in Supplementary Fig. S1.

Fibre tracking was conducted by the DSI Studio software (http://dsi-studio.labsolver.org). The DSI template, reconstructed by the generalised Q-sampling imaging method, was kindly provided by Drs. Wen-Yih Isaac Tseng and Yu-Jen Chen from the National Taiwan University Molecular Imaging Center and is now available as the original diffusion-weighted image on the NITRC website (https://www.nitrc.org/projects/ntu-dsi-122/). Detailed information of the NTU-122 template can be found in the work by Hsu *et al*.^43^. We used the streamline fibre-tracking algorithm in which both end regions of fibre tracking were defined as the complete meta-analytic image. This design preserved any fibre that originate and terminate within the meta-analytic image, regardless of direction and exact spatial location. The quantitative anisotropy threshold was set to 0.0085, and the angular threshold was set to 60°. To prevent the tracking process from being overwhelmed by short association fibres, we set a minimal length constraint of 20 mm. 150,000 fibres were traced out, and we applied hierarchical clustering to group the fibres into tracts and trimmed off scattered fibres. The spatial coordinates of fibre end points were clustered by the k-means clustering algorithm into network nodes. The resulting nodes were individually inspected to assess the clustering adequacy: If the algorithm erroneously clustered two ends of one tract into a single node due to spatial proximity, the node would be manually divided in two. This condition occasionally happened to fibres that straddled two gyri. This process gave rise to 34 nodes for the *dat* network.

The *anat* network—“*anat*” meaning “anatomical”—is more intuitive: It was built by tracing out major fibre connections between key anatomical landmarks involved in self-other processing. The purpose of building *anat* was to construct a self-other processing network in the conventional way; that is, atlas-dependent. Simulation result of *anat*—one of the two conventional networks—will be compared with that of *dat* in order to obtain insights into the influence construction approaches have on the dynamical properties.

The detailed construction process of *anat* is as follows. First, we manually traced out anatomical regions that were considered relevant to self-other processing in the earlier literature^5^,^7^,^27^,^28^,^29^. These regions included: Anterior cingulate cortex, posterior cingulate cortex, precuneus, medial prefrontal cortex, pars triangularis and pars opercularis of inferior frontal gyrus, temporo-parietal junction, and insula. We registered to the DSI template two different anatomical images—the normalised template in the Neurosynth package, and the MNI152 T1w brain image in the FSL package (http://fsl.fmrib.ox.ac.uk/fsl/fslwiki/)—as anatomical references, ensuring that the anatomical landmarks were accurately located and traced out. Streamline fibre tracking was used to trace out fibre connections between any two anatomical landmarks. Termination fibre count was set to 5,000 for all pairs, with a rescuing seed count of 500,000. The resulting *anat* network has 14 nodes.

Finally, the *anatpar* network—“*par*” meaning “partition”—was constructed by randomly segregating the 14 anatomical landmarks of *anat* into 34 segments, the same number as the node number of *dat*. In other words, *anatpar* had the same node number as *dat* and the same white matter structure as *anat*. As stated above, the main reason for building two conventional networks—*anat* and *anatpar*—was the lack of consensus on standard parcellation scheme. However, there was more to *anatpar* than merely another parcellation scheme. Earlier study suggested that node number or nodal scale might influence some network measures^44^; therefore, we matched the segment number of *anatpar* to the node number of *dat*. It is anticipated that comparing *dat* with two different versions of conventional networks—*anat* with anatomical nodes, *anatpar* with random nodes and matched node number—would make the comparison results more generalisable.

The null-hypothesis networks for *dat, anat* and *anatpar* were created by randomising these networks using the “randmio_und_connected” script in Brain Connectivity Toolbox^19^. 15 null-hypothesis networks were created for each of the three networks, preserving the degree distribution, connectivity strength and tract length.

### Dynamical neural systems

Dynamical systems were based on Kuramoto model of coupled oscillators. The mathematical derivation of Kuramoto model is detailed in the original work^33^. In brief, the model assumes global and weak coupling among local neuronal populations, whose activities are modelled as self-sustained oscillators. The behaviour of Kuramoto model is governed by the following equation:


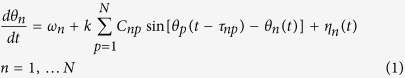


According to eq. (1), the instant angular velocity of a node *n* is determined by the intrinsic angular velocity *ω*_*n*_, the phase difference between node *n* and other nodes (*p*) connected to it by strength *C*_*np*_, and the noise *η*_*n*_. The phase differences were determined by the mean transmission time delays (τ_*np*_), which were linearly correlated with mean tract lengths between node *n* and other nodes (*p*). *C*_*np*_ is determined by the generalised fractional anisotropy (GFA) value. The *ω* values were randomly drawn from the gamma band (25 Hz to 75 Hz), whose correlation with global-level computation has been proposed previously^30^,^40^. To make the exploration of parameters as exhaustive as possible, 200 combinations of *ω* vectors—each composed of *N ω*_*n*_—were created for each neural network, yielding 200 dynamical systems. For the null-hypothesis networks, 40 sets of *ω* vectors were sampled for each randomised network. Because there is no consensus on the adjustment of *k*, the global scaling factor, it was arbitrarily set to 1,000 for an intermediate range of synchronisation levels. The *τ*_*np*_ values were divided by 20 and rounded to integers. Since our analysis depended on the stabilisation of synchronisation states, the noise term *η*_*n*_ was removed from the Kuramoto equation. Euler’s method was used in all integration processes, and all simulations were iterated through 2,000 steps to ensure stabilisation.

### Stroboscopic analysis

Stroboscopic analysis, which quantifies the synchronisation level between a pair of nodes, is part of the multistability evaluation process. Inspired by the theory of Poincare map^34^, this analysis takes into consideration some special cases that might be otherwise overlooked by other indices. The mathematical derivation of this synchronisation index is as follows:


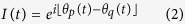



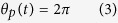



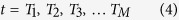



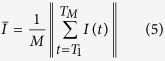


The basic concept is as follows: Suppose two nodes *p* and *q* produce two time-series signals, and these two signals can be mapped to the phase space in the form of *θ*_*p*_(*t*) and *θ*_*q*_(*t*). If *p* and *q* synchronise with each other, their phase difference should be bounded^35^. Under this condition, for *t* = *T*_*1*_, *T*_*2*_, *T*_*3*_…*T*_*M*_ when the phases of *p* equals 2π, the concurrent phases of *q* should be a fixed value. As illustrated in eqs (2) and (5), when the concurrent phase differences at these time-points are plotted on a unit circle in a polar coordinate system, the averaged norm of the sum of their corresponding complex unit vectors—the synchronisation index—will be 1. On the other hand, if *p* and *q* are completely uncorrelated, the concurrent phase differences will scatter around the unit circle, producing an index value of 0. In summary, stroboscopic analysis produces a synchronisation index for a pair of nodes generating continuous time-series signals, such as the nodes in Kuramoto dynamical system. This index value ranges from 1 for completely synchronised nodes to 0 for completely de-synchronised nodes.

In terms of implementing stroboscopic analysis with computer algorithm, oscillatory signals of the nodes produced by Kuramoto model were subjected to Hilbert transform to map them into phase space. The first 100 iterations of the time-series were deemed pre-stabilisation signals and discarded. These time-series were then analysed pairwisely. For every node pair, one of the two time-series served as the reference, and all the time-points where the reference phase approaches 2π were extracted. These time-points were then used to extract concurrent phases of another node, and unit vectors with these phase angles were averaged to obtain an index whose absolute value ranges from 0 to 1. The two nodes then reversed roles, yielding another index. The average of the two indices represented the synchronisation level of the two nodes.

It is noteworthy that our index takes into account not only in-phase synchronisation, but also synchronisation with various phase lags, including anti-phase synchronisation. It is different from another commonly used synchronisation index proposed by Shanahan^14^,^45^, which produces lower value as the phase lag between two signals increases. We did not assume that smaller phase lag indicates stronger synchronisation, as phase lag may be due to strong but distant signal transmission. Therefore, we devised this stroboscopic analysis index instead of adopting the index described above.

For demonstration in Fig. 5 the synchronisation patterns derived from the 20,000 simulation experiments (200 dynamical systems multiplied by 100 simulations) for each network were averaged and mapped back to the original *N* × *N* matrix, with nodes ordered as those in the GFA matrix. Auto-correlation values (i.e. main diagonal values) of these synchronisation matrices were set to 0.

### Elbow method for k-means clustering

We used the “gap” statistic to estimate the number of optimal clusters for k-means clustering of a dataset. A detailed description of the gap statistic can be found in the work of Tibshirani *et al*.^37^. Briefly, this method finds the cluster number at which the within-cluster error dispersion is the lowest compared to the expected value of error dispersion determined by Monte Carlo sampling from a reference distribution. The gap value is defined as follows:


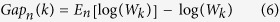



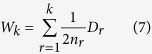


In eqs (6) and (7), *n* denotes the sample size, *k* denotes the number of clusters to be evaluated, *W*_*k*_ denotes the within-cluster error dispersion, *r* denotes the index of a cluster, and *n*_*r*_ denotes the number of data points in cluster *r*. In the case of k-means clustering, error dispersion is proportional to the sum of pairwise distances for all points within every individual cluster, denoted by *D*_*r*_ in the equation. The maximal cluster number to be evaluated was set to 6. The evaluation results were used to construct the “Clustered” matrix and correlation coefficient matrix for each dynamical system, as is illustrated in Fig. 3. The number distributions of synchronisation states presented in Fig. 4 were obtained by pooling all optimal *k* values of dynamical systems belonging to each category. The optimal *k* values of all 15 null-hypothesis networks for each network category were also pooled for analysis.

### Software

Neurosynth meta-analysis was done by Python version 2.7. Kuramoto model simulation, stroboscopic analysis and the k-means algorithm with Elbow method were done using Matlab, 2014b version. Visualization of activation map and anatomical correlates in Fig. 1 was done by the Caret software (http://www.nitrc.org/projects/caret/)^46^.

## Additional Information

**How to cite this article:** Chen, Y.-A. and Huang, T.-R. Multistability of the Brain Network for Self-other Processing. *Sci. Rep.* **7**, 43313; doi: 10.1038/srep43313 (2017).

**Publisher's note:** Springer Nature remains neutral with regard to jurisdictional claims in published maps and institutional affiliations.

## Supplementary Material

Supplementary Information
