# Supplementary material for: Multistability of the Brain Network for Self-other Processing
Source: Sci Rep. 2017 Mar 3;7:43313. doi: 10.1038/srep43313 (PMC5335699; doi:10.1038/srep43313)
Supplement: Supplementary Information [file srep43313-s1.pdf]

Supplementary Information

Multistability of the Brain Network for Self-other Processing.

Yi-An Chen[1], Tsung-Ren Huang[1,*]

[1]Department of Psychology, National Taiwan University, 10617 Taipei, Taiwan

[*]E-mail address of corresponding author: tren@mil.psy.ntu.edu.tw

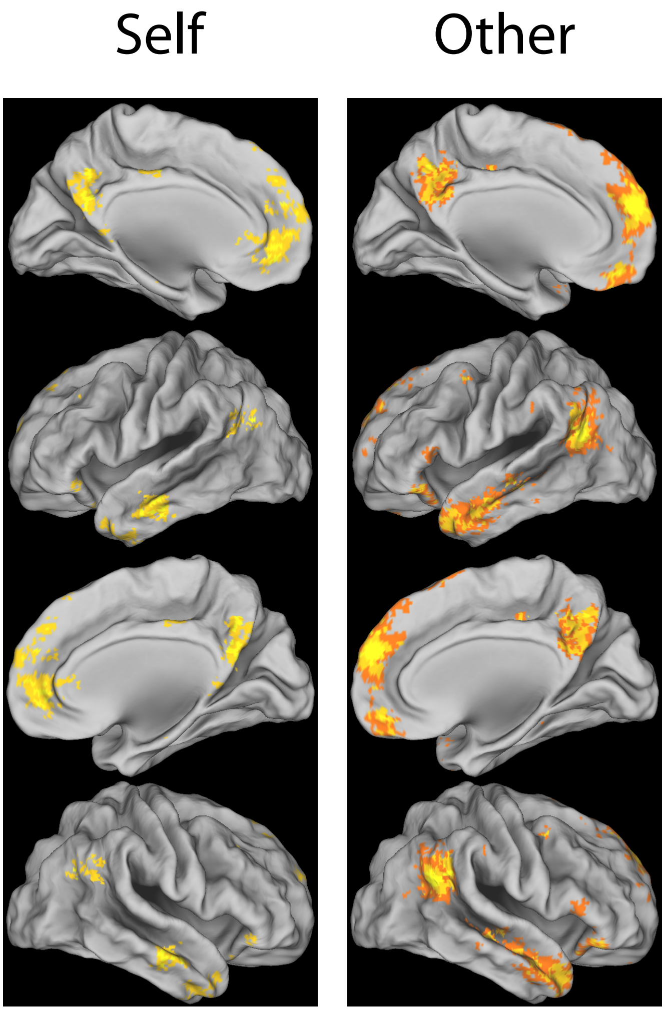

Supplementary figure S1. Meta-analytic images of self-related and other-related processing areas. The "self" image was created by Neurosynth term-based meta-analysis with the key word "self referential," the "other" image was created by the combination of "mind tom," "theory mind," "mentalizing" minus "self." The two groups of studies used to create these images were contrasted by the two-way chi-square analysis tool of Neurosynth to identify coordinates whose frequencies of being reported differed between the two groups; FDR was set to 0.05. No statistically different coordinates were identified.

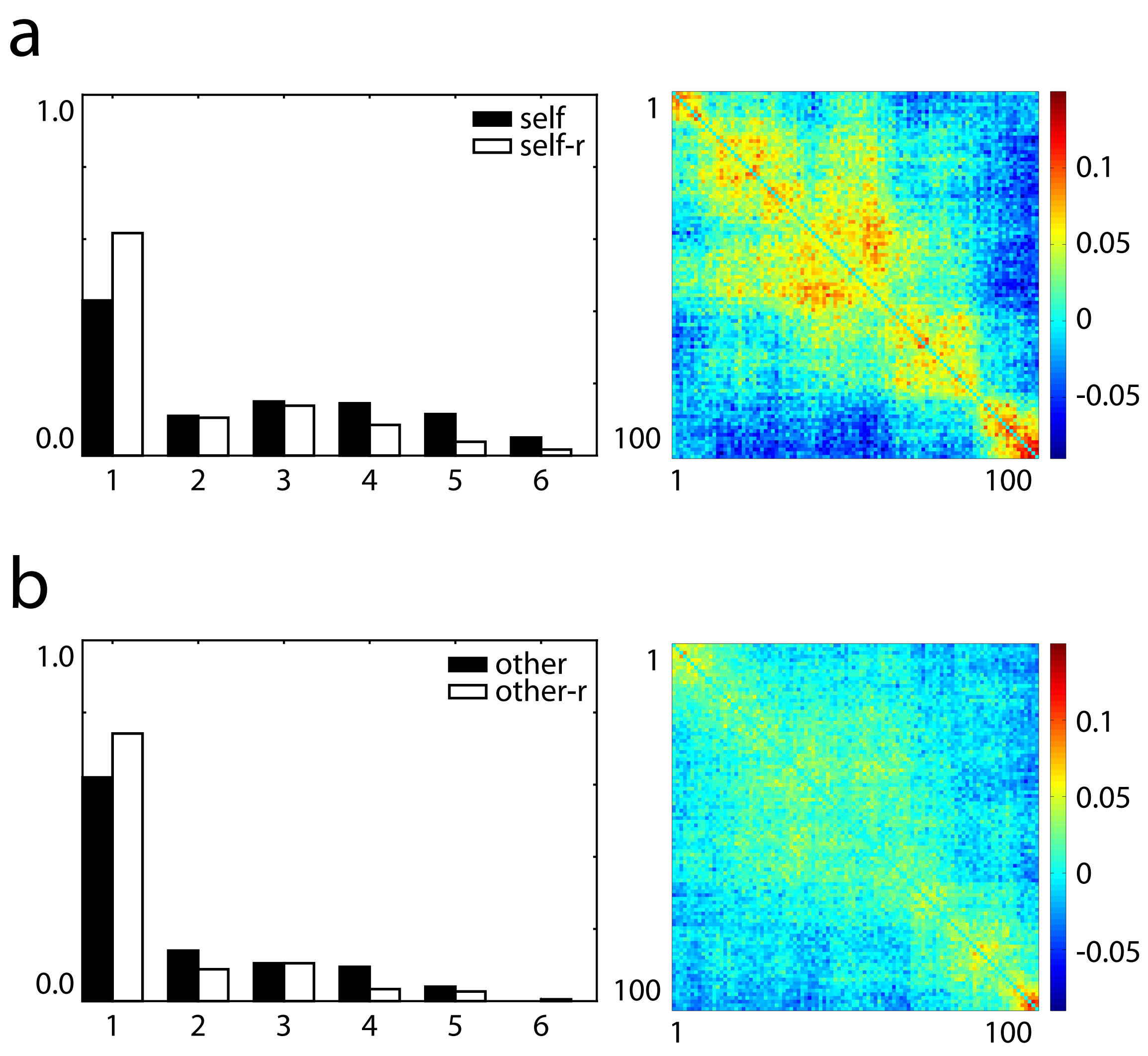

Supplementary figure S2: Multistability of the "self" network and "other" network. (a) Left: Distributions of synchronisation state numbers of *self* and *self-r* showed statitically significant difference ($p = 4.6247 \times 10^{-5}$). Right: Difference between average *self* and *self-r* correlation matrices showed aggregaion of high-value entries near the main diagonal, suggesting multistability. (b) Left: Distributions of synchronisation state numbers of *other* and *other-r* did not present statistically significant difference ($p = 0.0215$). Right: Difference between average *other* and *other-r* correlation matrices did not show obvious aggregation of high-value entries around the main diagonal.
